# A large-scale screening and functional sorting of tumour microenvironment prognostic genes for breast cancer patients

**DOI:** 10.3389/fendo.2023.1131525

**Published:** 2023-03-01

**Authors:** Bo Xiao, Mingwei Li, Mingxuan Cui, Chengliang Yin, Bo Zhang

**Affiliations:** ^1^ Key Laboratory of Immune Microenvironment and Disease (Ministry of Education), Tianjin Key Laboratory of Cellular and Molecular Immunology, Department of Immunology, School of Basic Medical Sciences, Tianjin Medical University, Tianjin, China; ^2^ Faculty of Medicine, Macau University of Science and Technology, Macau, Macau SAR, China

**Keywords:** breast cancer, miRNA, tumor microenvironment, prognostic model, cell proliferation

## Abstract

**Purpose:**

The aim of this study was to systematically establish a comprehensive tumour microenvironment (TME)-relevant prognostic gene and target miRNA network for breast cancer patients.

**Methods:**

Based on a large-scale screening of TME-relevant prognostic genes (760 genes) for breast cancer patients, the prognostic model was established. The primary TME prognostic genes were selected from the constructing database and verified in the testing database. The internal relationships between the potential TME prognostic genes and the prognosis of breast cancer patients were explored in depth. The associated miRNAs for the TME prognostic genes were generated, and the functions of each primary TME member were investigated in the breast cancer cell line.

**Results:**

Compared with sibling controls, breast cancer patients showed 55 differentially expressed TME prognostic genes, of which 31 were considered as protective genes, while the remaining 24 genes were considered as risk genes. According to the lambda values of the LASSO Cox analysis, the 15 potential TME prognostic genes were as follows: ENPEP, CCDC102B, FEZ1, NOS2, SCG2, RPLP2, RELB, RGS3, EMP1, PDLIM4, EPHA3, PCDH9, VIM, GFI1, and IRF1. Among these, there was a remarkable linear internal relationship for CCDC102B but non-linear relationships for others with breast cancer patient prognosis. Using the siRNA technique, we silenced the expression of each TME prognostic gene. Seven of the 15 TME prognostic genes (NOS2, SCG2, RGS3, EMP1, PDLIM4, PCDH9, and GFI1) were involved in enhancing cell proliferation, destroying cell apoptosis, promoting cell invasion, or migration in breast cancer. Six of them (CCDC102B, RPLP2, RELB, EPHA3, VIM, and IRF1) were favourable for maintaining cell invasion or migration. Only two of them (ENPEP and FEZ1) were favourable for the processes of cell proliferation and apoptosis.

**Conclusions:**

This integrated study hypothesised an innovative TME-associated genetic functional network for breast cancer patients. The external relationships between these TME prognostic genes and the disease were measured. Meanwhile, the internal molecular mechanisms were also investigated.

## Introduction

Breast cancer is a rapidly growing public health problem of global concern (1). In developed countries, breast cancer has become the second most common cause of cancer death in women and is also the leading cause of cancer death in women in low- and middle-income countries (2). Several elements have been shown to be closely associated with breast cancer, such as hormone-related elements, pregnancy-related elements, anthropometric index elements, physical condition, dietary elements, and environmental exposures (1). Clinically, breast cancer patients can be classified into different subtypes. To date, classifications have been based on different expression patterns of progesterone receptor (PR), estrogen receptor (ER), and human epidermal growth factor receptor-2 (HER-2). Traditional classification does not fully reflect the heterogeneity of breast cancer. For this purpose, breast cancer could also be divided into five different subtypes according to genetic profiling, which represent the heterogeneity of breast cancer (3). Clinically, the recommended diagnostic methods for breast cancer patients are MRI, mammography, ultrasonography, and PET (2). Among them, the recommended technique for the diagnosis of breast cancer is mammography, which, to date, represents the gold standard screening method for breast cancer patients (4). However, approximately 20% of breast cancers are missed by mammography (5). When mammography fails to detect breast cancer, ultrasound and magnetic resonance imaging can be used to detect breast cancer (3).

Tumour progression has long been thought to be associated with epigenetic changes in tumour cells (6). There is increasing evidence that the cells and matrix components surrounding tumour cells also play an indispensable role in the tumour process. Together, these components constitute the tumour microenvironment (TME) (7). The TME consists of various TME-related genes localised or secreted by immune cells and stromal cells, which play a critical role in tumour proliferation and metastasis (8). For example, some immune cells in the TME, such as M1 macrophages, have a significant effect on tumour inhibition by activating Th1 responses. At the same time, other cells such as tumour-associated endothelial cells, cancer-associated fibroblasts, and M2 macrophages in the TME can promote tumour growth and proliferation (9) (10). In addition, localised stromal cells, immune cells, and tumour cells in the TME can interact with each other through cytokines, which could effectively accelerate tumour growth and proliferation. For example, integrins and soluble factors (e.g., IL-6, SDF1, and HGF) could mediate the interaction between tumour cells and the stroma. Signalling pathways involving MAPK, PI3K/Akt, ERK1/2, and STAT have been shown to be highly activated in tumour cells. On the other hand, the activities of anti-apoptotic proteins (e.g., Bcl-2 and Bcl-XL) could be enhanced in the TME, which then initiate cancer development (11).

The miRNAs (short for microRNAs) are a super-family of small non-coding RNAs, most of which are approximately 21 nucleotides in length. Although these miRNAs cannot be translated, they can regulate transcription by binding to the 5’ or 3’ non-coding regions of mRNA (12). The miRNAs play an important role in tumour development and are thought to be key factors in TME. The miRNAs can affect tumour progression by regulating the cell cycle, manipulating programmed cell death, controlling cell invasion, and targeting angiogenesis (13).

Since TME has received more and more attention in cancer research, we have thoroughly analysed the expression of 760 TME-relevant prognostic genes and their prognostic values in breast cancer patients. In addition to the broad screening, the internal functions of each primary TME prognostic gene for breast cancer prognosis were also thoroughly investigated in this integrated study.

## Materials and methods

### Data source

We used two independent data sources in this study. The breast cancer patients from the TCGA-BRCA database were considered as a constructing database. At the same time, the breast cancer patients from the GSE162228 chip were treated as a testing database.

For the constructing database, we downloaded the gene information profile and corresponding clinical information of breast cancer patients from The Cancer Genome Atlas (TCGA, https://tcga-data.nci.nih.gov/tcga/) database, which contains 1,082 breast cancer patients with complete survival information. In addition, for the testing database, we downloaded a dataset from the GEO database (GEO, https://www.ncbi.nlm.nih.gov/geoprofiles/?term=GSE162228), which contains 109 breast cancer patients with complete survival information. The genetic information of the breast cancer patients in the GEO dataset was analysed using the Affymetrix Human Genome U133A Array platform.

### TME-relevant prognostic gene identification and selection

In this study, the identification and selection of TME-relevant prognostic genes followed the previously established protocol (14). Essentially, the complete list of genes was obtained from 10 published studies providing transcriptomic signatures for multiple immune and stromal cell populations (15–25).

The final differentially expressed TME-relevant prognostic genes for breast cancer patients were selected and verified in two steps: first, a single-factor Cox regression analysis for breast cancer prognosis was developed to analyse the expression values of each TME-relevant prognostic gene in breast cancer samples, with a threshold of *p*< 0.05; second, bootstrapping was performed to test the genes that passed the initial filtering for robustness as follows: 70% of patients randomly selected from the cohort were tested for the survival impact of their genes.

### LASSO Cox regression analysis

To avoid over-fitting the model, redundant prognosis-related molecules were removed from the dimension using LASSO Cox regression analysis. The model’s penalty parameter (lambda value) was also determined using 10-fold cross-validation, with the smallest lambda value selected to remove redundant prognosis-related molecules.

For this purpose, the glmnet function package of the R language was performed for LASSO Cox regression analysis to further infer the TME prognostic genes related to breast cancer prognosis. The following formula was established to calculate the risk score for each individual breast cancer sample:


$$\text{Risk Score}=\sum_{\text{i}=1}^{\text{n}}{\text{Coef}}_{\text{i }}{*\text{X}}_{\text{i}}$$

In this formula, Coefi represents the risk coefficient of each factor estimated by the LASSO-Cox model. X_i_ indicates the expression activity of each TME prognostic gene. The breast cancer patients of the constructing database and the testing database could be subdivided into a high-risk group and a low-risk group based on the median of the corresponding calculated risk score.

### Survival analysis

The survival and survminer packages in the R language were used to analyse the survival of each patient. The package was based on the Kaplan–Meier method (26).

### Prediction of miRNA targets

The targets of potential miRNAs were achieved *via* miRBase (www.http://www.mirbase.org), TargetScan (http://www.targetscan.org), miRanda (http://www.microrna.org/microrna/home.do), miRWalk (http://www.ma.uni-heidelberg.de/apps/zmf/mirwalk), TarBase (http://diana.cslab.ece.ntua.gr/tarbase), and miRecords (http://mirecords.biolead.org).

### Cell culturing

The mda-MB-453 cell line, purchased from ATCC Co., was selected as a breast cancer cell line in this study and cultured in L15 culturing medium with 10% fetal bovine serum plus 1% P/S.

### Luciferase reporter assay

The 3’ non-coding region of each TME gene was synthesised and inserted into the XhoI and NotI sites of the pCheck2 reporter luciferase vector downstream of the luciferase gene. The wild type (WT) or mutant plasmid and negative control or miRNA were transfected together into mda-MB-453 cells. Final luciferase analysis was performed using a dual luciferase reporter analysis system (Promega, USA).

### Tumour manner analysis of mda-MB-453 cells

The corresponding TME prognostic gene siRNAs (50 nmol/L) (OriGene) and the corresponding negative control transfection were obtained by Lipofectamine-2000 (Sigma-Aldrich, USA).

The siRNA-transfected cells were harvested and the proliferation ability was measured using the CCK-8 method (Fisher, China). At the same time, the apoptosis ability of the cells was examined by the method of flow cytometry after Annexin V FITC/PI double staining. For transwell migration assays, 2.5×10^4^ cells were seeded in the appropriate serum-free medium into the pre-coated upper chamber. The 500-μl complete medium was used as chemoattractant in the lower chambers. The incubation time was set at 48 h, after which cells without migration or invasion were removed. The final number of migrated or invaded cells was examined using Image-Pro Plus version 6.0 software. All experiments were repeated three times independently.

### Statistical analysis

The multi-factor Cox regression model was built to analyze whether risk score could predict the survival of patients with breast cancer independently of all other factors. The statistical analysis was established by R software, with version number v4.2.2.

## Results

### TME prognostic gene selections for breast cancer prognosis

First, the breast cancer patients from the TCGA-BRCA database were used as a constructing database. We focused on TME genetic profiling in breast cancer patients. The selection scope was based on a complete list of 760 TME genes, which are listed in **Supplementary Table S1**. Differential analysis was performed using single-factor Cox regression analysis. The 760 TME-relevant prognostic gene expression values were treated as continuous variables in the regression analysis. The 55 differentially expressed TME prognostic genes were finally screened out as *p*-value< 0.05 (**Table 1**).

**Table 1:** The Univariate Cox analysis results for differentially expressed TME prognostic genes.

Univariate Cox analysis results			
Gene Symbol	HR (95% CI for HR)	*p*-value	*z*
ENPEP	1.66 (1.28–2.16)	0.000139074	3.81
CCDC102B	2.33 (1.44–3.75)	0.000522431	3.47
FEZ1	1.99 (1.33–2.98)	0.000855644	3.33
NOS2	1.94 (1.31–2.88)	0.000958565	3.3
SCG2	1.24 (1.09–1.41)	0.00128473	3.22
RPLP2	0.75 (0.63–0.90)	0.001925764	-3.1
RELB	0.73 (0.59–0.90)	0.003646782	-2.91
RGS3	1.76 (1.20–2.58)	0.003899135	2.89
EMP1	1.32 (1.09–1.60)	0.005084147	2.8
PDLIM4	0.78 (0.65–0.93)	0.005222158	-2.79
EPHA3	1.26 (1.07–1.49)	0.005598623	2.77
PCDH9	1.46 (1.11–1.93)	0.006761393	2.71
VIM	0.72 (0.57–0.92)	0.007530978	-2.67
GFI1	0.58 (0.39–0.87)	0.008598062	-2.63
IRF1	0.77 (0.64–0.94)	0.008833897	-2.62
EGR3	0.84 (0.73–0.96)	0.009926168	-2.58
SERPING1	0.78 (0.64–0.94)	0.010483576	-2.56
GPR157	1.32 (1.06–1.63)	0.011500637	2.53
EMP3	0.75 (0.59–0.94)	0.012977354	-2.48
NOVA2	1.87 (1.13–3.09)	0.014175508	2.45
APOD	0.91 (0.84–0.98)	0.01471526	-2.44
GZMB	0.84 (0.72–0.97)	0.014940485	-2.43
FBLN5	0.78 (0.64–0.95)	0.015242065	-2.43
LAMB3	0.86 (0.77–0.97)	0.015320158	-2.42
EGR2	0.81 (0.68–0.97)	0.020596099	-2.32
PLAT	0.88 (0.79–0.98)	0.02066215	-2.31
C1R	0.81 (0.68–0.97)	0.021310129	-2.3
ST3GAL1	1.20 (1.03–1.41)	0.021409383	2.3
NHSL2	1.48 (1.06–2.06)	0.021435989	2.3
RDX	1.30 (1.04–1.63)	0.021922109	2.29
PSPC1	0.69 (0.51–0.95)	0.024554127	-2.25
IGFBP6	0.82 (0.69–0.98)	0.028380367	-2.19
BHLHE41	0.84 (0.71–0.98)	0.028445965	-2.19
HSD17B6	1.34 (1.03–1.74)	0.030702069	2.16
ZBED2	0.71 (0.52–0.97)	0.030961087	-2.16
PTGER3	0.86 (0.75–0.99)	0.031050691	-2.16
PCDH17	1.47 (1.03–2.08)	0.032445543	2.14
RHOC	0.78 (0.62–0.98)	0.032588416	-2.14
HIVEP2	1.35 (1.02–1.78)	0.032782401	2.13
GLIPR1	0.70 (0.50–0.97)	0.03279175	-2.13
LAMC2	0.88 (0.79–0.99)	0.0329582	-2.13
ANXA5	0.71 (0.51–0.98)	0.035117301	-2.11
ACTG2	0.90 (0.82–0.99)	0.035668501	-2.1
TNFSF4	1.32 (1.02–1.72)	0.036985692	2.09
SFPQ	0.71 (0.52–0.98)	0.037613255	-2.08
EDIL3	1.15 (1.01–1.32)	0.037647622	2.08
PAFAH1B1	1.39 (1.02–1.89)	0.037714152	2.08
PLCL1	0.80 (0.65–0.99)	0.039631052	-2.06
TM4SF4	1.30 (1.01–1.68)	0.041774483	2.04
NGFR	0.85 (0.73–1.00)	0.044023122	-2.01
CAPG	0.83 (0.69–1.00)	0.045120591	-2
NID2	1.25 (1.00–1.55)	0.045980878	2
PAICS	1.24 (1.00–1.53)	0.047716607	1.98
NCAPD3	1.28 (1.00–1.63)	0.049061581	1.97
CTSZ	0.80 (0.63–1.00)	0.049850603	-1.96

In the list, the genes with HR value less than 1 were favourable for breast cancer prognosis (protective TME prognostic genes). On the other hand, the genes with HR value greater than 1 were unfavourable for breast cancer prognosis (risk TME prognostic genes). For this purpose, 31 of the 55 genes were considered as protective genes, while the remaining 24 genes were considered as risk genes.

**Figure 1:**
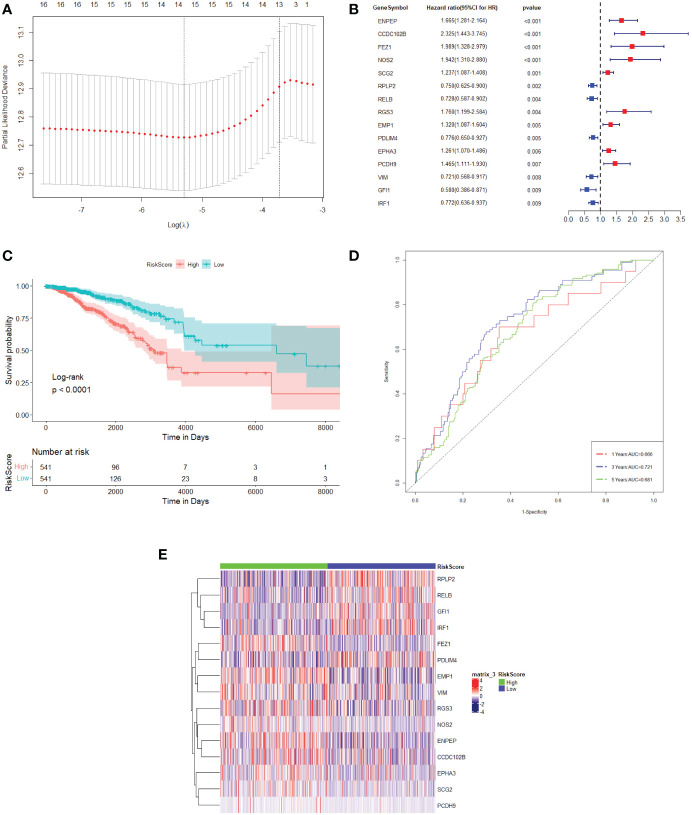
TME prognostic gene selections for breast cancer prognosis. **(A)** The plot of the determination of the tuning parameter lambda in the LASSO regression model. The horizontal axis is the log (lambda) and the vertical axis is the partial likelihood deviation value. The lambda value corresponding to the smallest value is the best. **(B)** Forest plot of the 15 most significant TME prognostic genes associated with breast cancer prognosis. HR is the hazard ratio and 95% CI is the 95% confidence interval. **(C)** Kaplan–Meier survival curve in the TCGA dataset. The horizontal axis represents time, while the vertical axis represents survival. Different colours represent different groups. The *p*-value is based on the log-rank test. **(D)** The time-dependent ROC curve. The horizontal axis is the false-positive rate, while the vertical axis is the true-positive rate. The accuracy of prediction is assessed by the AUC (area under the ROC curve) value. **(E)** The expression heat map of the 15 TME prognostic genes selected from the TCGA dataset.

To remove redundant prognosis-related molecules, the 55 differentially expressed TME prognostic genes were then plotted in a LASSO Cox regression analysis. As shown in **Figure 1A**, the optimal number of differentially expressed TME prognostic genes was determined to be 15 (**Figure 1A**, with the lowest lambda value). Therefore, the forest plot of the top 15 genes with the smallest *p*-value among the 55 genes is shown in **Figure 1B**. The 15 TME prognostic genes were ENPEP, CCDC102B, FEZ1, NOS2, SCG2, RPLP2, RELB, RGS3, EMP1, PDLIM4, EPHA3, PCDH9, VIM, GFI1, and IRF1.

The expression values of 15 TME prognostic genes were then weighted with the regression coefficients of the LASSO Cox regression model to generate a risk score for predicting survival in breast cancer patients. Each patient’s risk score was calibrated individually. Using the risk score as a further selection criterion, the 1,082 patients were divided into a high-risk group and a low-risk group based on the median of the risk score. According to the survival analysis, the patients in the high-risk sample showed an overall poor survival rate compared to the low-risk sample (**Figure 1C**).

In addition, it could be shown that the AUC of the 1-year, 3-year, and 5-year survival period of the breast cancer patients were 0.666, 0.721, and 0.681, respectively, obtained from the time-dependent ROC (**Figure 1D**). These results indicated that the risk model could accurately predict the prognosis of breast cancer patients. Meanwhile, the 15 TME prognostic genes were remarkably different when comparing the high-risk and low-risk groups (**Figure 1E**), which further confirmed the specificity of the 15 TME prognostic genes as well as the efficiency of the risk score constructed by them.

### Verification of potential TME prognostic genes and risk score using the testing database

**Figure 2:**
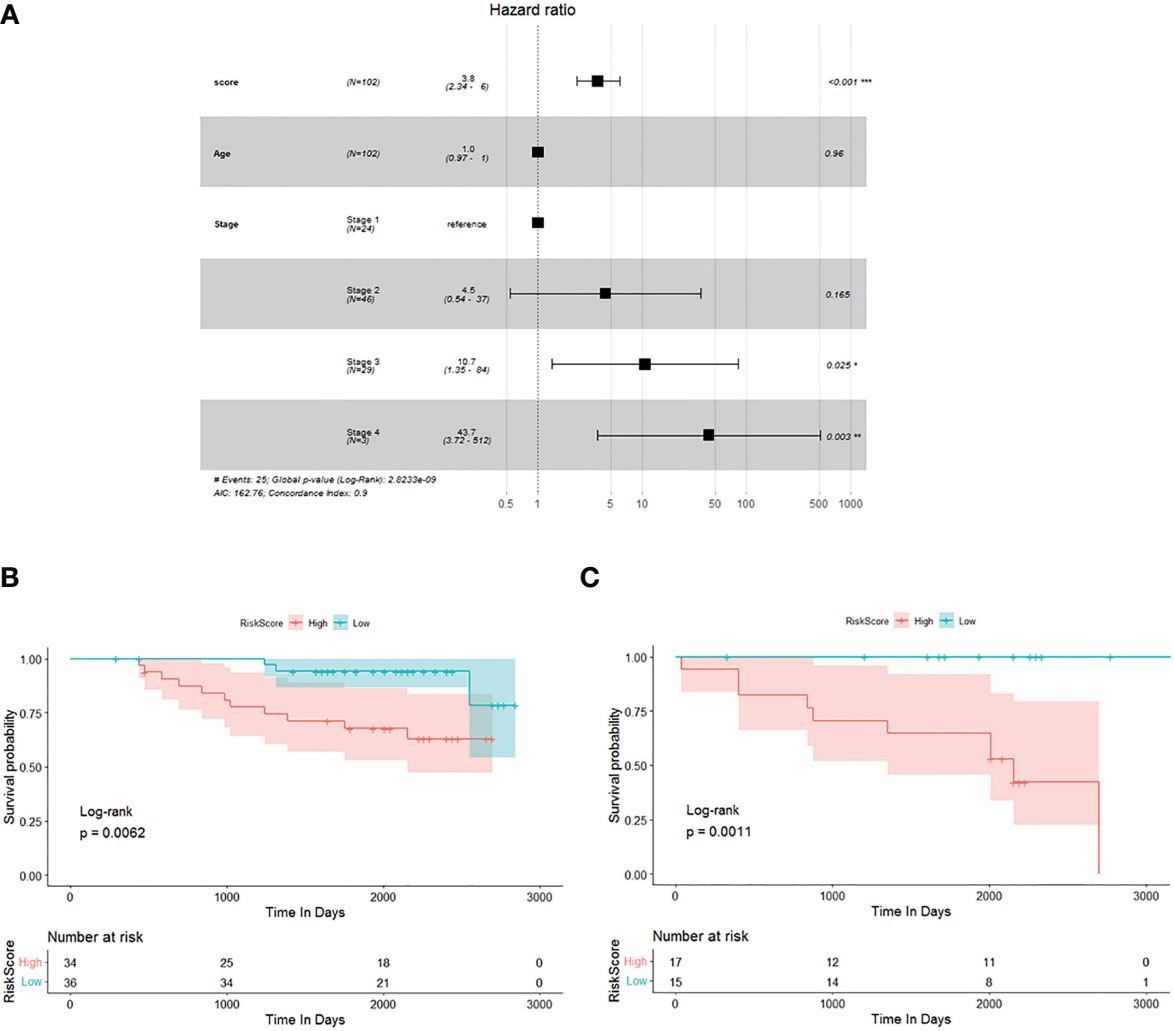
Verification of potential TME prognostic genes and risk score using testing database. **(A)** Multivariate Cox regression analysis forest plot. **(B, C)** The Kaplan–Meier survival curve of breast cancer patients<60 years old and ≥60 years old, respectively.

Previously, we selected primary TME prognostic genes from the constructing database and obtained the corresponding risk factor using the TME prognostic genes. We then sought to confirm the results using an independent testing database (GSE database). Out of 109 breast cancer patients (stage 0 patients were excluded) with complete clinical information, 102 were processed for the study. The age, TNM stage, and risk score of each individual breast cancer patient were all subjected to multivariate Cox regression analysis to decide whether the risk score was an independent prognostic indicator for breast cancer patients in the test database. As shown in **Figure 2A**, it could be demonstrated that the risk score was dramatically associated with the overall survival of the testing database, and the samples with a high risk score had a higher risk of death and were unfavourable for prognosis (HR = 3.8, 95% CI: 2.34–6, *p*< 0.001).

To investigate the prognostic value of the risk score established by 15 potential TME-relevant prognostic genes, we further regrouped patients from the testing database and performed Kaplan–Meier survival analysis. Patients were divided into group A (**Figure 2B**, <60 years old) and group B (**Figure 2C**, ≥60 years old). Patients were defined as high risk or low risk based on the risk score determined by 15 potential TME-relevant prognostic genes. Regardless of age, patients in the high-risk group had a significantly lower overall survival rate than those in the low-risk group (**Figures 2B, C**). These results concluded that the risk score constructed by primary TME prognostic genes was an independently accurate indicator for predicting the prognosis of breast cancer patients. Furthermore, the risk factor was shown to be independent of TNM stage (**Supplementary Figure 1**).

### Correlation between potential selected TME prognostic genes and prognosis of breast cancer patients

**Figure 3:**
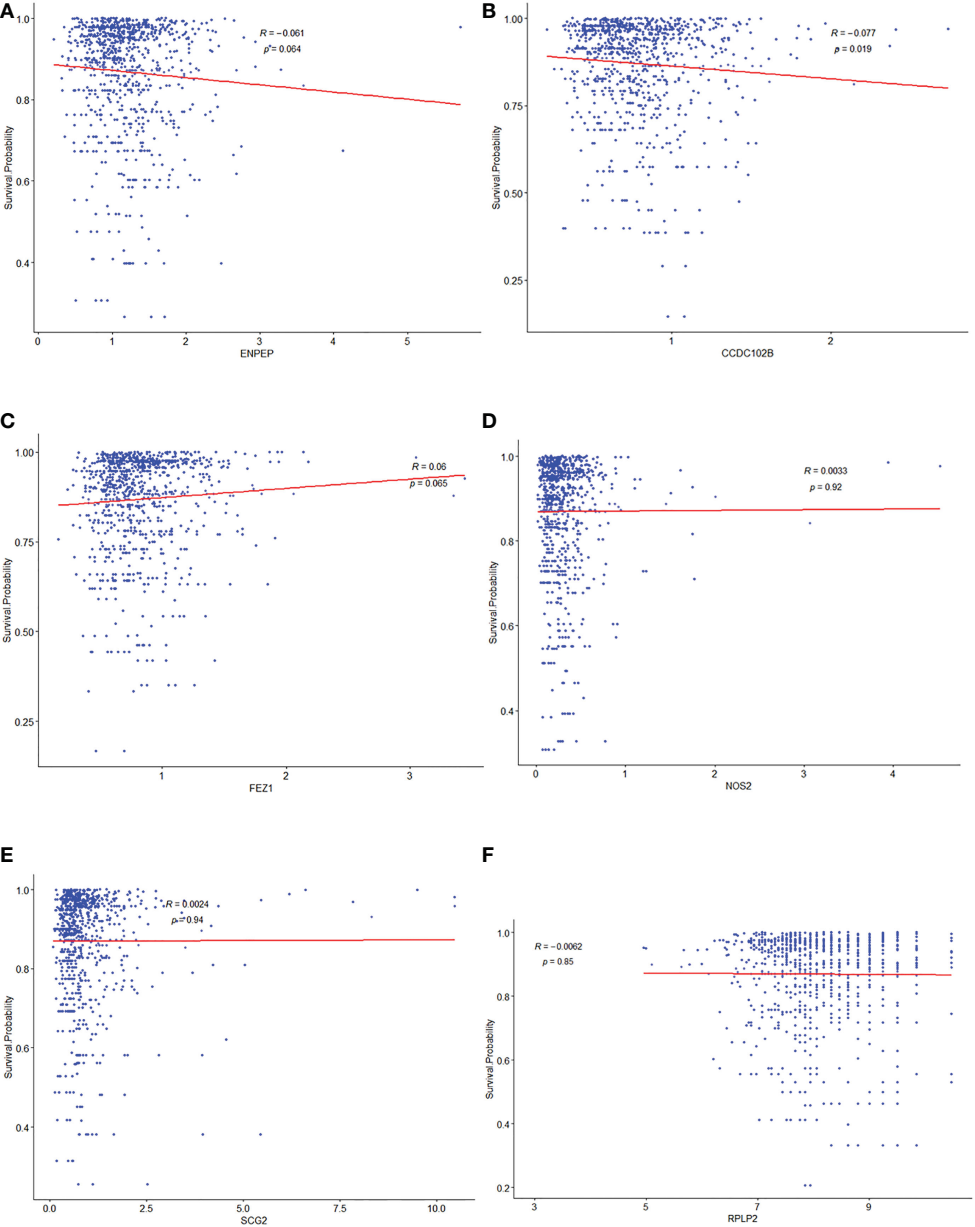
Correlationship between potential selected TME prognostic genes and prognosis of breast cancer patients. The relationship analysis between prognosis of breast cancer patients and ENPEP **(A)**, CCDC102B **(B)**, FEZ1 **(C)**, NOS2 **(D)**, SCG2 **(E)**, and RPLP2 **(F)**, respectively.

To investigate the relationships between 15 TME prognostic genes and the prognosis of breast cancer patients in depth, the expressions of these TME prognostic genes were re-entered into the regression model with the survival probability of each individual patient from the construction database. As shown in **Figure 3**, the risk TME prognostic genes were negatively associated with prognosis (**Figures 3A–E**, ENPEP, CCDC102B, FEZ1, NOS2, and SCG2), while the protective TME prognostic genes were positively associated with prognosis (**Figure 3F**, RPLP2). The remaining TME prognostic genes are shown in **Supplementary Figure 2**. Based on the results of forest plot (**Figure 1B**) and regression model analysis (**Figure 3**), ENPEP, CCDC102B, FEZ1, NOS2, SCG2, RGS3, EMP1, EPHA3, and PCDH9 were suggested as risk TME prognostic genes. In contrast, RPLP2, RELB, PDLIM4, VIM, GFI1, and IRF1 were suggested as protective TME prognostic genes.

Among them, there was an apparent linear internal relationship for CCDC102B (**Figure 3B**), but non-linear relationships for others (**Figures 3A, C–F**).

### Prediction of potential miRNAs for TME prognostic gene of breast cancer prognosis

The 15 TME prognostic genes and associated potential target miRNAs were searched using six well-established search tools, namely, miRBase, TargetScan, miRanda, miRWalk, TarBase, and miRecords. The search results were sorted according to TarGetScore (the complete list is shown in **Table 2**).

**Table 2:** The list of TarGetScore for TME-associated miRNAs.

Symbol	miRNA	Expectation score
EPHA3	hsa-miR-559	99.59458
EPHA3	hsa-miR-548a-5p	99.36135273
EPHA3	hsa-miR-548ab	99.36135273
EPHA3	hsa-miR-548ad-5p	99.36135273
EPHA3	hsa-miR-548ae-5p	99.36135273
EPHA3	hsa-miR-548ak	99.36135273
EPHA3	hsa-miR-548am-5p	99.36135273
EPHA3	hsa-miR-548ap-5p	99.36135273
EPHA3	hsa-miR-548aq-5p	99.36135273
EPHA3	hsa-miR-548ar-5p	99.36135273
EPHA3	hsa-miR-548as-5p	99.36135273
EPHA3	hsa-miR-548au-5p	99.36135273
EPHA3	hsa-miR-548ay-5p	99.36135273
EPHA3	hsa-miR-548b-5p	99.36135273
EPHA3	hsa-miR-548bb-5p	99.36135273
EPHA3	hsa-miR-548c-5p	99.36135273
EPHA3	hsa-miR-548d-5p	99.36135273
EPHA3	hsa-miR-548h-5p	99.36135273
EPHA3	hsa-miR-548i	99.36135273
EPHA3	hsa-miR-548j-5p	99.36135273
EPHA3	hsa-miR-548o-5p	99.36135273
EPHA3	hsa-miR-548w	99.36135273
EPHA3	hsa-miR-548y	99.36135273
PCDH9	hsa-miR-7-1-3p	97.86567
PCDH9	hsa-miR-7-2-3p	97.86567
SCG2	hsa-miR-3143	97.55045
EPHA3	hsa-miR-3163	97.42054
VIM	hsa-miR-708-3p	97.16357
PCDH9	hsa-miR-5688	97.03809
PCDH9	hsa-miR-520d-5p	96.82901
PCDH9	hsa-miR-524-5p	96.82901
PCDH9	hsa-miR-3973	96.6787
ENPEP	hsa-miR-4427	96.52265
EPHA3	hsa-miR-551b-5p	95.62846
EPHA3	hsa-miR-5692a	95.48851
PCDH9	hsa-miR-495-3p	95.3761
VIM	hsa-miR-4328	94.28759
EPHA3	hsa-miR-627-3p	94.25463
EPHA3	hsa-miR-4307	94.19724
EPHA3	hsa-miR-520d-5p	94.1869
EPHA3	hsa-miR-524-5p	94.1869
SCG2	hsa-miR-8485	93.95476
EPHA3	hsa-miR-3059-5p	93.88569
SCG2	hsa-miR-548aj-3p	93.04963
SCG2	hsa-miR-548x-3p	93.04963
SCG2	hsa-miR-4801	92.84489
PCDH9	hsa-miR-4801	92.36807
SCG2	hsa-miR-5692a	92.20465
PDLIM4	hsa-miR-619-3p	91.75736
RELB	hsa-miR-3059-5p	91.64647
PCDH9	hsa-miR-4731-3p	91.57227
PCDH9	hsa-miR-153-5p	91.43969
ENPEP	hsa-miR-3163	91.25889
FEZ1	hsa-miR-4474-3p	90.7065
EPHA3	hsa-miR-4474-3p	90.55867
VIM	hsa-miR-124-3p	90.54101
VIM	hsa-miR-506-3p	90.54101
PCDH9	hsa-miR-510-3p	90.54088
SCG2	hsa-miR-4731-3p	90.48228
ENPEP	hsa-miR-551b-5p	90.42114
PCDH9	hsa-miR-4427	90.38796
ENPEP	hsa-miR-3662	90.21936
SCG2	hsa-miR-95-5p	90.12317
FEZ1	hsa-miR-373-5p	89.94208
FEZ1	hsa-miR-616-5p	89.94208
FEZ1	hsa-miR-371b-5p	89.94208
PCDH9	hsa-miR-4699-3p	89.43566
EPHA3	hsa-miR-510-3p	89.2435
GFI1	hsa-miR-4777-5p	88.6116
EPHA3	hsa-miR-9902	88.42069
PCDH9	hsa-miR-4474-3p	88.3186
PCDH9	hsa-miR-627-3p	88.16555
IRF1	hsa-miR-12136	88.04969
EPHA3	hsa-miR-153-5p	87.93277
ENPEP	hsa-miR-4777-5p	87.771
PCDH9	hsa-miR-95-5p	87.66373
EPHA3	hsa-miR-373-5p	86.72648
EPHA3	hsa-miR-616-5p	86.72648
EPHA3	hsa-miR-371b-5p	86.72648
PCDH9	hsa-miR-4778-5p	86.6743
SCG2	hsa-miR-5688	86.6212
RGS3	hsa-miR-6500-3p	86.09
EMP1	hsa-miR-548n	86.03104672
ENPEP	hsa-miR-641	85.99173
ENPEP	hsa-miR-3617-5p	85.99173
VIM	hsa-miR-548n	85.9015
ENPEP	hsa-miR-4778-5p	85.8444
CCDC102B	hsa-miR-153-5p	85.66093
NOS2	hsa-miR-559	85.5333
GFI1	hsa-miR-5688	85.4781
SCG2	hsa-miR-495-3p	85.3661
SCG2	hsa-miR-7-1-3p	85.3661
SCG2	hsa-miR-7-2-3p	85.3661
PDLIM4	hsa-miR-9902	85.33963
CCDC102B	hsa-miR-3163	84.9453
PCDH9	hsa-miR-3617-5p	84.7735
CCDC102B	hsa-miR-548aj-3p	84.51465939
CCDC102B	hsa-miR-548x-3p	84.51465939
PCDH9	hsa-miR-551b-5p	83.75769
GFI1	hsa-miR-3973	83.6984
PCDH9	hsa-miR-8485	83.6918
PDLIM4	hsa-miR-7111-5p	83.1606
PDLIM4	hsa-miR-4723-5p	82.8831
PDLIM4	hsa-miR-5698	82.8831
PDLIM4	hsa-miR-6870-5p	82.8831
EMP1	hsa-miR-4699-3p	82.7515
EPHA3	hsa-miR-6500-3p	82.61788
SCG2	hsa-miR-9902	82.6176
VIM	hsa-miR-12136	82.6027
GFI1	hsa-miR-495-3p	82.5314
NOS2	hsa-miR-548a-5p	82.3559
NOS2	hsa-miR-548ab	82.3559
NOS2	hsa-miR-548ad-5p	82.3559
NOS2	hsa-miR-548ae-5p	82.3559
NOS2	hsa-miR-548ak	82.3559
NOS2	hsa-miR-548am-5p	82.3559
NOS2	hsa-miR-548ap-5p	82.3559
NOS2	hsa-miR-548aq-5p	82.3559
NOS2	hsa-miR-548ar-5p	82.3559
NOS2	hsa-miR-548as-5p	82.3559
NOS2	hsa-miR-548au-5p	82.3559
NOS2	hsa-miR-548ay-5p	82.3559
NOS2	hsa-miR-548b-5p	82.3559
NOS2	hsa-miR-548bb-5p	82.3559
NOS2	hsa-miR-548c-5p	82.3559
NOS2	hsa-miR-548d-5p	82.3559
NOS2	hsa-miR-548h-5p	82.3559
NOS2	hsa-miR-548i	82.3559
NOS2	hsa-miR-548j-5p	82.3559
NOS2	hsa-miR-548o-5p	82.3559
NOS2	hsa-miR-548w	82.3559
NOS2	hsa-miR-548y	82.3559
EMP1	hsa-miR-708-3p	82.153
PCDH9	hsa-miR-641	82.0849
CCDC102B	hsa-miR-4723-5p	82.0752
CCDC102B	hsa-miR-5698	82.0752
CCDC102B	hsa-miR-7111-5p	82.0752
CCDC102B	hsa-miR-6870-5p	82.0752
GFI1	hsa-miR-4328	81.86927
PCDH9	hsa-miR-3143	81.8312
PCDH9	hsa-miR-619-3p	81.5165
EPHA3	hsa-miR-124-3p	80.9182
EPHA3	hsa-miR-506-3p	80.9182
ENPEP	hsa-miR-510-3p	80.66176
EPHA3	hsa-miR-3662	80.33439
PCDH9	hsa-miR-4307	80.0189

**Figure 4:**
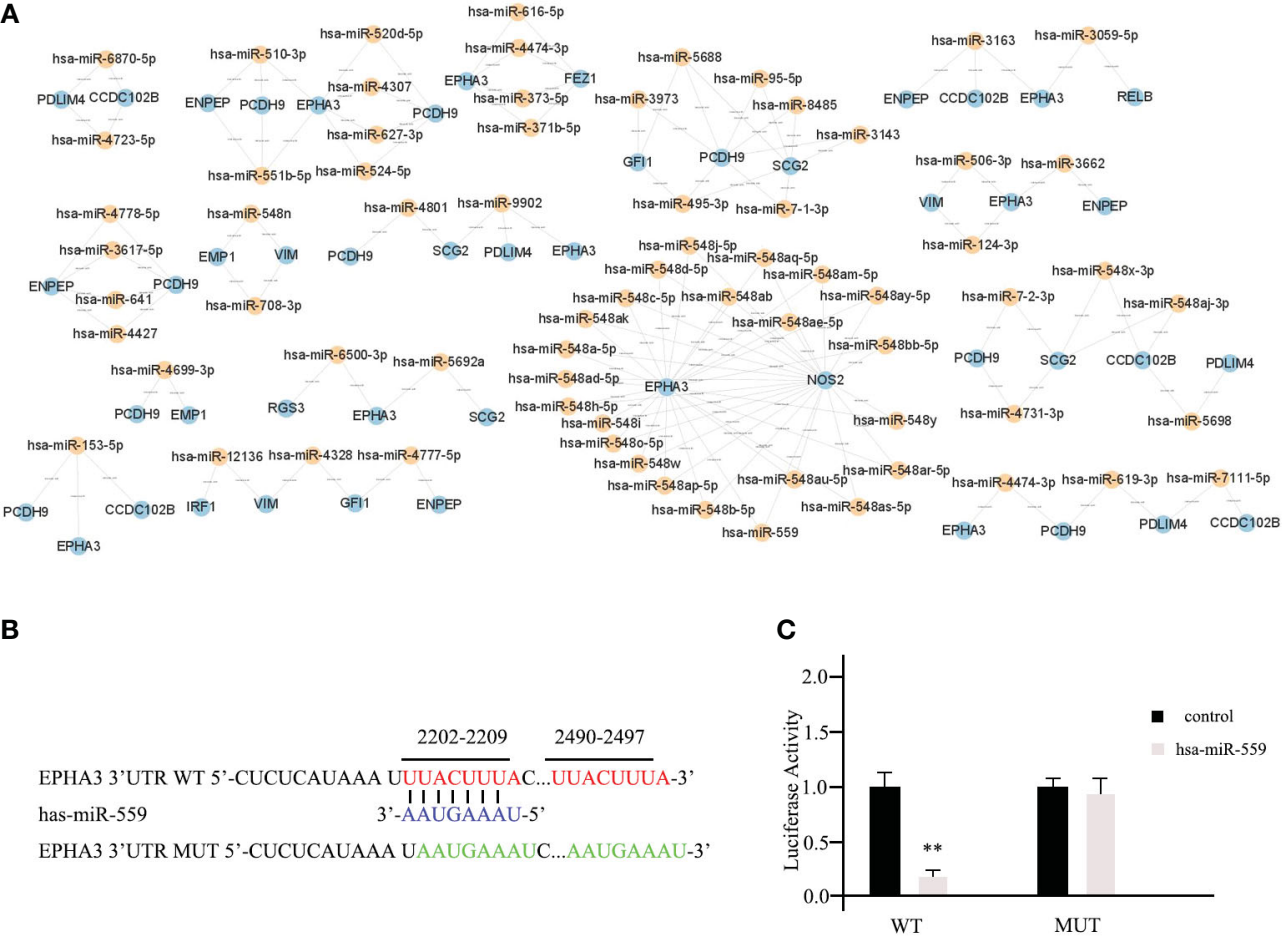
Prediction of potential miRNAs for TME prognostic gene of breast cancer prognosis. **(A)** The establishment of a network of 15 TME prognostic genes and targeting miRNAs using the miRNAs with TarGetScore scores greater than 80. **(B)** The schematic depicting the wild type (WT) and the mutant (MUT)-specific fragments of 3’ UTR of EPHA3. **(C)** The luciferase reporter assays results for hsa-miR-559 in breast cancer cells. ** as an indication of P < 0.01 compared with internal control.

In the list, the higher the score, the higher the confidence. The primary miRNAs with prediction scores above 80 were considered relatively reliable, while those with prediction scores below 60 were considered less reliable. The final network of 15 TME prognostic genes and targeting miRNAs was constructed using all primary miRNAs with prediction scores greater than 80 (**Figure 4A**). Notably, all miRNAs with high scores interacted with EPHA3, suggesting that the miRNA-manipulated signalling cassette may play a pivotal role in the functions of this TME prognostic gene.

To further validate our predictive results, the direct interactions between potential miRNAs and targeted TME prognostic genes were supported by the luciferase reporter assay. As shown in **Figure 4B**, the WT-specific fragments of the 3’ UTR of EPHA3 and mutant (MUT) were cloned into the luciferase reporter. Subsequently, hsa-miR-559 (with the highest predictive score for EPHA3) and non-targeting miRNA control were transfected into mda-MB-453 cells. According to the luciferase reporter assay results, hsa-miR-559 could directly bind to the 3’UTR of EPHA3 and inhibit its function (WT; **Figure 4C**). On the other hand, the inhibition was significantly reduced when the sequence of the identified interaction site was mutated (mutant; **Figure 4C**).

### Diverse functions of TME prognostic genes for breast cancer development

To date, it has been suggested that promoting cell proliferation, destroying cell apoptosis, promoting cell invasion, and promoting cell migration are the primary causes of cancerogenesis. Based on these hypotheses, we sought to investigate the functions of 15 TME prognostic genes. We used siRNA technology to knock down the expression of each TME prognostic gene. Knockdown of risk TME prognostic genes (ENPEP, CCDC102B, FEZ1, NOS2, SCG2, RGS3, EMP1, EPHA3, and PCDH9) could induce decreased cell proliferation, increased cell apoptosis, and impaired cell invasion or migration. In contrast, deletion of protective TME prognostic genes (RPLP2, RELB, PDLIM4, VIM, GFI1, and IRF1, shown in dark colour in each graph) showed the opposite pattern.

**Figure 5:**
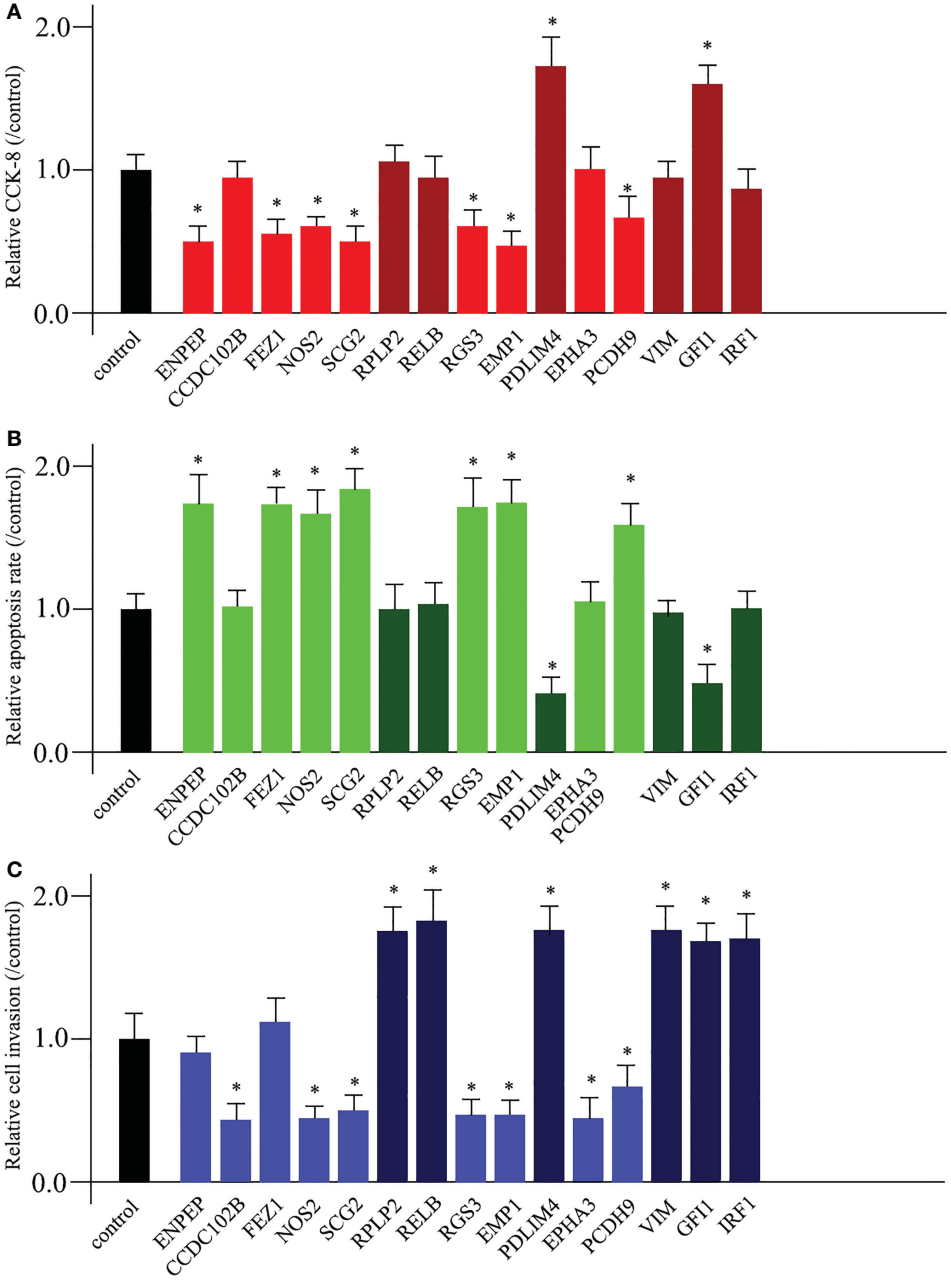
Diverse functions of TME prognostic genes for breast cancer development. The cell proliferation analysis **(A)**, the cell apoptosis examination **(B)**, and the cell invasion or migration measurement **(C)** comparing untreated control and 15 TME prognostic gene siRNA-transfected breast cancer cells. * as an indication of P < 0.05 compared with internal control.

Among the 15 TME prognostic genes, most of them (NOS2, SCG2, RGS3, EMP1, PDLIM4, PCDH9, and GFI1) were involved in all three entries, while 6 of them (CCDC102B, RPLP2, RELB, EPHA3, VIM, and IRF1) were favourable for maintaining cell invasion or migration. Furthermore, only two of them (ENPEP and FEZ1) were favourable for the processes of cell proliferation and apoptosis (**Figures 5A–C**).

## Discussion

The TME generally refers to the non-cancerous cells and various components present in the tumour, consisting of immune cells and stromal cells as well as molecules (27). The constant connections between tumour cells and TME components play a pivotal role in tumour initiation, progression, development, and metastasis (28). Functionally, the TME components could harbour tumour cells through direct interaction with surrounding cells *via* the lymphatic and circulatory systems to ultimately influence cancer development (8). Thus, the TME has served as a potential therapeutic target for cancer treatment and has attracted basic research and clinical interest (29). In breast cancer, various preclinical and clinical studies have provided ample evidence that TME genes are involved not only in breast cancer progression but also in determining therapeutic response (30). Furthermore, some of the TME genes have shown a prominent value for the existing predictive and prognostic marker panels. The significant alterations in the TME genes could be recognised as a critical element in the development of breast cancer.

From the 760 TME-relevant prognostic genes, we narrowed down our selection to 15 potential TME prognostic genes with a notable differential expression pattern in breast cancer patients. Among these, we proposed ENPEP, CCDC102B, FEZ1, NOS2, SCG2, RGS3, EMP1, EPHA3, and PCDH9 as risk TME prognostic genes. RPLP2, RELB, PDLIM4, VIM, GFI1, and IRF1 were identified as protective TME prognostic genes (**Figure 2**). As every coin has two sides, the protective TME factors from stromal cells or immune cells could abolish cancer cell metastasis or facilitate the immune system defence mechanism. On the other hand, risk TME factors from suppressive immune cells, together with the extracellular matrix (ECM) element, could work together to exhibit anti-tumour immunity and promote breast cancer development. Either enhanced risk TME gene expression or suppressed protective TME prognostic gene expression, caused by dysfunctional or aberrant specific signalling pathways, could contribute to the development of breast cancer. However, there are some limitations to this study. For example, due to the complicated and dynamic status of TME, it is quite difficult to show the exact expression for each individual gene in TME. Our identification of TME-relevant prognostic genes in breast cancer patients was based on previous established literature searches and summaries. At the same time, we did not claim that these genes are only functional in TME, and we only focused on their functional transition from TME to tumour progression. In the future, it may be useful to show where (which cell types) and how (the expression level) the TME-relevant prognostic gene is expressed in the breast cancer environment.

Despite the many breakthroughs in cancer research, there is still a lack of solid evidence for the cancer process due to the extremely complicated molecular mechanisms that underlie the disorders. In breast cancer, we prefer the “seed-and-soil” hypothesis, which suggests that the primary localised breast cancer cells represent “seed” cells (31). The permissive secondary tissues could be considered as “soil” for migrative or invasive cancer cells. The permissive secondary tissues could be considered as “soil” for migrative or invasive cancer cells. Based on this hypothesis, the TME could be considered as a “fertiliser” consisting of ECM, various inflammatory cytokines, and other remodelling enzymes (32). The cancer cells themselves interact with the surrounding functions and components of the TME to initiate or suppress cancer development. Our parallel data demonstrated that CCL, a critical chemokine (C-C) motif ligand of the TME, exerts both anti-cancer properties dependent on the recruitment of anti-cancer tumour-infiltrating lymphocytes (TILs), which destroy cancer cells, and pro-cancer functions correlating with the recruitment of cells functional to stimulate tumour growth, enhance tumour cell migration, and block the activities of tumour suppressors (article in preparation). These were similar to previously published data (33–35).

**Figure 6:**
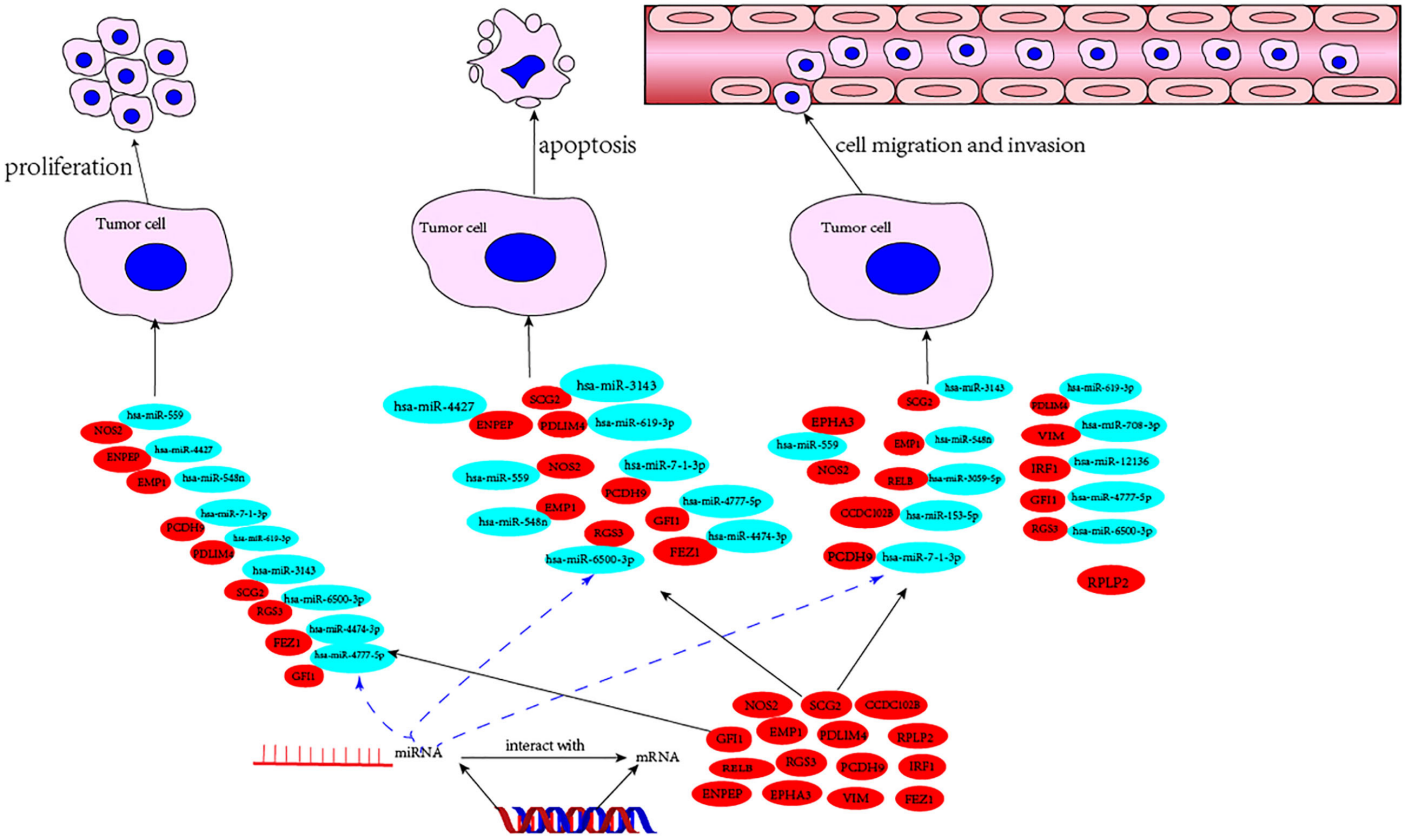
The mechanism diagram of potential TME prognostic genes and associated miRNAs for breast cancer progression.

Using a broad screening approach, we identified 15 attractive TME genes. Furthermore, we testified the functions of each individual TME from three aspects, namely, cell proliferation, cell apoptosis, and cell invasion or migration manipulation. From the results, the enhancement of cell proliferation and the attenuation of cell apoptosis could be closely related, and none of the TME prognostic genes showed a single function for them. Therefore, cell proliferation and cell apoptosis could be combined in this study. As expected, seven of the TME prognostic genes (NOS2, SCG2, RGS3, EMP1, PDLIM4, PCDH9, and GFI1) showed dual functions in breast cancer progression. The rest had only a single function in breast cancer development (**Figure 6**).

Some of the potential members are “old hands” in breast cancer research. For example, ENPEP has been shown to be a key factor involved in breast cancer cell proliferation through the function of inducing G2/M cell cycle arrest and reducing anchorage-independent cell growth of mammary origin (36). Si and colleagues claimed that coiled-coil domain containing 102B (CCDC102B) was apparently increased in metastatic lesions in lymph nodes of breast cancer patients (37). Increased expression of CCDC102B was required for breast cancer metastasis. Here, the functions of CCDC102B may be achieved through the regulation of NF-κB pathway components. The FEZ1 gene was mapped to chromosome 8p22 (a common aberration in human tumours). Mutations in the FEZ1 gene have been found in a variety of cancers (38). EMP1, which stands for epithelial membrane protein gene 1, together with EMP2 and EMP3 belong to the PMP22 (peripheral myelin protein 22-kDa) gene family. There are six members of this gene family, namely, brain cell membrane protein 1, MP20, EMP1, EMP2, EMP3, and PMP22. In lung cancer, EMP1 has been implicated as a biomarker for gefitinib resistance. EMP1, EMP2, and EMP3 have been reported as novel therapeutic targets in human cancer (39). Previously, the PDLIM4 gene was identified as a tumour suppressor. In breast cancer cells, the PDLIM4 gene encodes an adaptor protein that functions as a key regulator of stress fibre assembly, actin cytoskeleton remodelling, and epithelial–mesenchymal transition (40). The IRF1 gene has been shown to have essential functions during the epithelial–mesenchymal transition process. However, IRF1 is also required for the maintenance of epithelial differentiation. This dual role of IRF1 is context-dependent, particularly for the modulation of epithelial–mesenchymal plasticity, which may be of interest for future breast cancer treatment (41).

Overall, we have identified several interesting TME prognostic genes involved in breast cancer progression through a large-scale screening approach. The targeted miRNAs and the molecular mechanisms highlighted were validated both *in vivo* and *in vitro*, opening a new window for future studies.

## Data availability statement

The datasets presented in this study can be found in online repositories. The names of the repository/repositories and accession number(s) can be found below: https://www.ncbi.nlm.nih.gov/, GSE162228.

## Author contributions

Conception and design: BZ. Administrative support: CY and BZ. Provision of study materials: BX and CY. Collection and assembly of data: BX, ML and MC. Data analysis and interpretation: ML. All authors contributed to the article and approved the submitted version.

## Supplementary material

Supplementary Table 1 The entire list of 760 TME-relevant prognostic genes.

Supplementary Figure 1 The Kaplan-Meier survival curve of breast cancer patients with stage TNM I-II **(A)** and TNM III-IV **(B)** respectively.

Supplementary Figure 2 Correlationship between potential selected TME prognostic genes and prognosis of breast cancer patients. The relationship analysis between prognosis of breast cancer patients and RELB **(A)**, RGS3 **(B)**, EMP1 **(C)**, PDLIM4 **(D)**, EPHA3 **(E)**, PCDH9 **(F)**, VIM **(G)**, GFI1 **(H)** and IRF1 **(I)**.
