# Supplementary figures and images for: A large-scale screening and functional sorting of tumour microenvironment prognostic genes for breast cancer patients
Source: Front Endocrinol (Lausanne). 2023 Mar 1;14:1131525. doi: 10.3389/fendo.2023.1131525 (PMC10014861; doi:10.3389/fendo.2023.1131525)

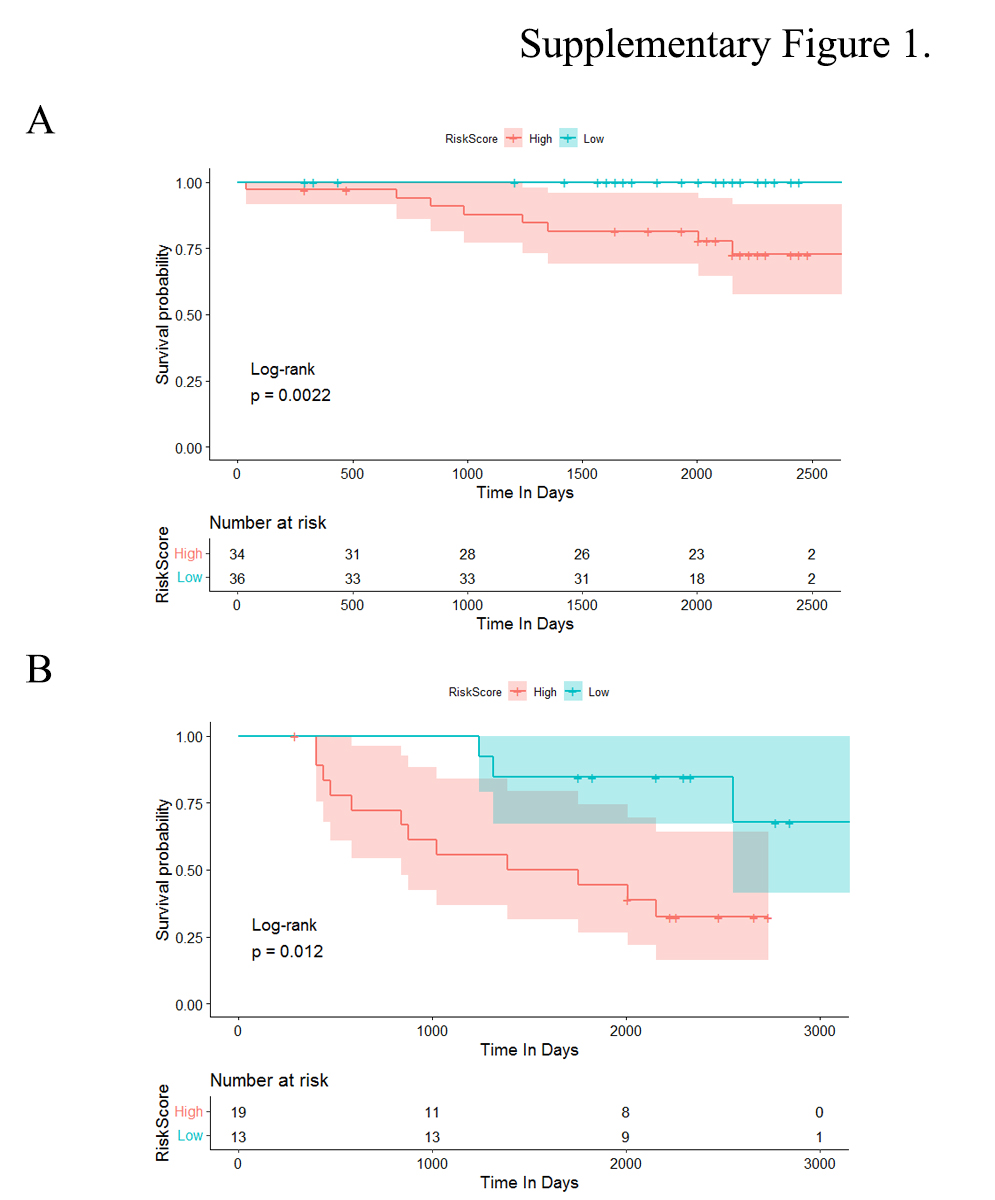

Supplement: Supplementary file 1 [file Image_1.jpeg]

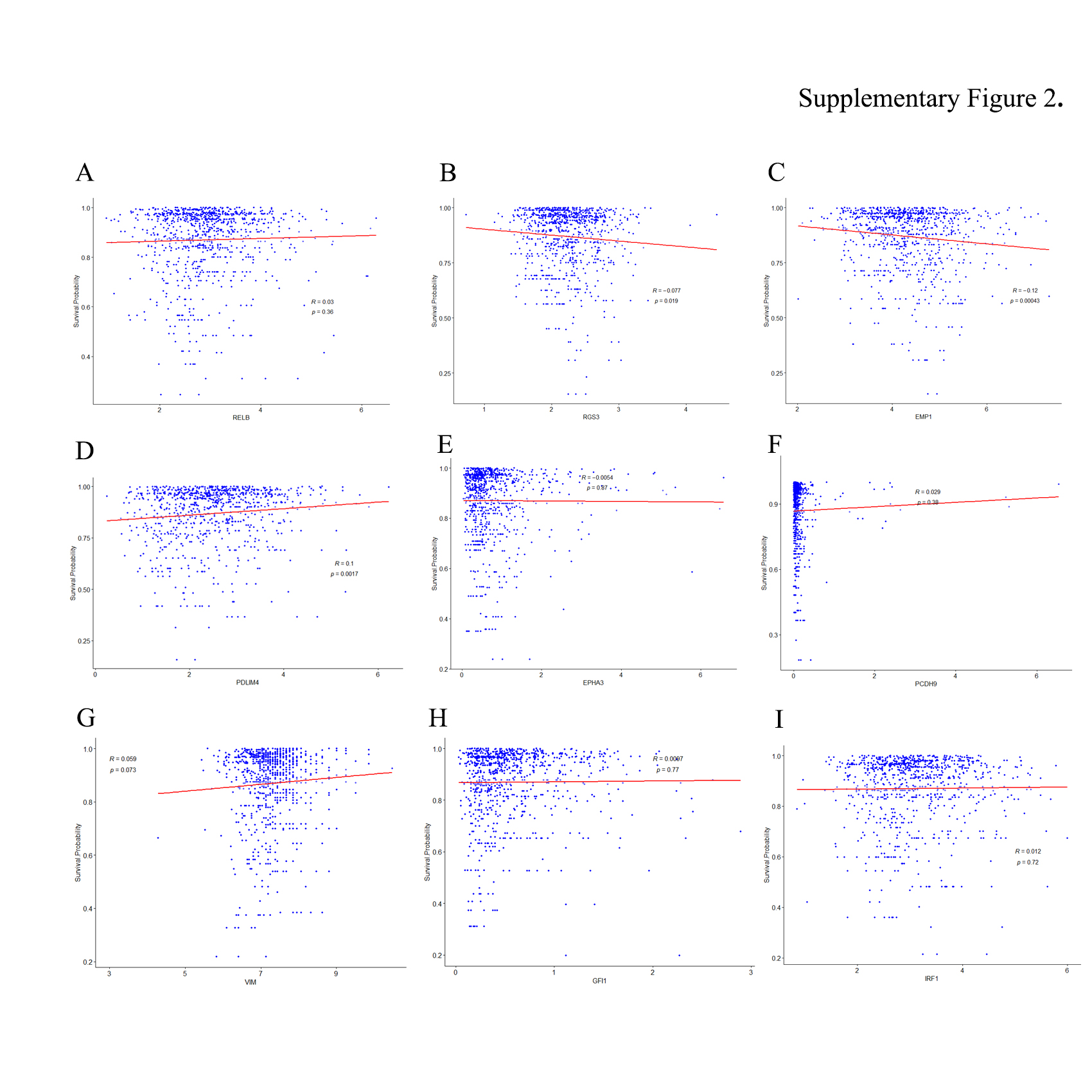

Supplement: Supplementary file 2 [file Image_2.jpeg]
